# Can social cognition be a mediator in mother-infant interaction? Description of a series of cases

**DOI:** 10.1192/j.eurpsy.2026.11930

**Published:** 2026-07-31

**Authors:** A. Roca Lecumberri, B. Vilaseca, A. Torres, E. Roda, E. Sole, E. Gelabert

**Affiliations:** 1 Perinatal Mental Health Unit, Hospital Clínic Barcelona; 2 Department of Clinical and Health Psychology, Universitat Autonoma de Barcelona, Barcelona, Spain

## Abstract

**Introduction:**

The scientific literature has reported alterations in social cognition (SC) and in mother-infant interaction (MII) in women with severe mental disorders. However, despite the importance of SC, few studies have examined its relationship with early MII in this population.

**Objectives:**

To describe a case series of women with diagnostis of severe mental illness and explore the possible association between alterations in SC domains and MII.

**Methods:**

Cross-sectional evaluation of a case series of postpartum women diagnosed with bipolar disorder, schizoaffective disorder or schizophrenia. A baseline assessment was performed to collect socio-demographic data, reproductive health information, and personal and family psychopathological histories. The PANSS was administered for psychotic disorders, and the HDRS and YMRS were administered for bipolar disorders. The

assessment also included a neuropsychological evaluation using the WAIS-IV vocabulary subtest, WCST, and TMT-B, as well as an

evaluation of the three domains of social cognition: theory of mind, emotional processing, and attributional biases. A second assessment was performed using the Care Index to evaluate mother-baby interaction.

**Results:**

Seventeen women were included in the study, with a mean age of 39 years. The diagnoses were bipolar disorder (n=13), schizoaffective disorder (n=3), and one mother diagnosed with schizophrenia.

Difficulties were found in the social cognition (SC) domains, specifically Theory of Mind and emotional intelligence (EI). Regarding MII, low scores were observed in maternal sensitivity, responsiveness, and infant participation. A significant correlation was found between both measures.

**Conclusions:**

The findings of difficulties in certain SC and MII domains are consistent with the current literature. Future research could explore these differences and their potential impact on MII. These results cannot be explained by active symptoms of the

disorder

**Disclosure of Interest:**

A. Roca Lecumberri Grant / Research support from: Instituto de Salud Carlos III . PI23/01352 (PI049327), B. Vilaseca: None Declared, A. Torres: None Declared, E. Roda: None Declared, E. Sole: None Declared, E. Gelabert: None Declared

